# The Effectiveness of an Intervention to Promote Awareness and Reduce Online Risk Behavior in Early Adolescence

**DOI:** 10.1007/s10964-015-0401-2

**Published:** 2015-12-24

**Authors:** Janneke D. Schilder, Marjolein B. J. Brusselaers, Stefan Bogaerts

**Affiliations:** Department of Developmental Psychology, School of Social and Behavioral Sciences, Tilburg University, 5000 LE Tilburg, The Netherlands; Department of Criminal Law and Criminology, Catholic University of Leuven, Leuven, Belgium; Belgian Cybercrime Centre of Excellence for Training, Research and Education, Leuven, Belgium

**Keywords:** School-based intervention, Internet, Online risk awareness, Online risk behavior, Early adolescence

## Abstract

The current study explored the effect of a school-based intervention on online risk awareness and behavior in order to shed light on a relatively unexplored field with high practical relevance. More than 800 Belgium primary school children (grade 4 and 6) were assessed at two measurements (*n* T1 = 812, 51.2 % female; *n* T2 = 819, 51.3 % female) before and after the intervention. Half of them received a 10 min classroom intervention indicating online risks. Children in the control group received a 10 min presentation concerning online applications without any emphasis on risks. Children in the intervention group were more likely to be aware of online risks directly after the intervention; this effect was still noticeable 4 months after. Reporting of online risk behavior in the intervention group was also higher compared to the control group who did not receive the intervention. Overall online risk awareness and online risk behavior were negatively associated and the awareness did not modulate the association between the intervention and online risk behavior. Furthermore, individual differences were assessed. Girls were more likely to be aware of online risks and asserted less online risk behavior than boys were. In line with the imperative in adolescence to become more risk taking, children in a higher grade were more likely to behave in a risky manner when online. The current study provides a valuable starting point for further research on how to decrease online risk behavior in early adolescence.

## Introduction

The current generation is the first that takes the existence of the Internet for granted. The EU Kids Online Survey (Livingstone and Haddon 2009) reports that children of 9–16 years old go online for on average 88 min per day. However, rather positive labels to this generation like “whiz kids” disguise the potential negative site of this increase in Internet use. All day access to the Internet via a computer, smartphone or tablet might expose these children to several dangers. Reports, such as the EU kids online report, also emphasize the risky side of their Internet behavior (Livingstone and Haddon 2009). However, merely knowing the risks does not change the fact that caregivers feel empty handed when dealing with new technologies that did not exist when they were young themselves (Livingstone 2009). The current study will therefore examine the online risk behavior and online risk awareness in early adolescents and examine whether an awareness raising intervention can be used to change this to the better.

De Moor et al. (2008) provide a classification of three different categories of risks that children can encounter when they are on the Internet. The first category is content risk. This category includes different kind of risks concerning possible harmful contents, for example websites showing naked or porn images. More than half of the teenagers accidently saw porn websites when surfing on the Internet (De Moor et al. 2008). Although not all children experienced negative effects when they face sexual pictures on the Internet, one quarter of the children were extremely upset (Mitchell et al. 2014). Other examples of risks in the content category are violence or racism websites, but they also refer to a lack of critical skills of children to judge the reliability of information that they see on the Internet (De Moor et al. 2008; for an overview of negative effects also see Valcke et al. 2011).

The second category named by De Moor et al. (2008) is contact risk. Risks in this category all refer to activities on the Internet toward known or unknown persons. Examples include the disclosure of personal information such as the home address or phone number, cyber bullying, and chatting in risky settings. An example of the latter is that 16.1 % of the children indicated that they were asked to give sexual information about themselves, and 10.6 % even received a question to perform a sexual action for another person (De Moor et al. 2008). The third category is commercial risk. This category refers to the acts of commercial organizations that focus on the exploitation of Internet users (De Moor et al. 2008). Examples are the abuse of personal information and spam.

A mere part of the literature concerning online risk behavior can be classified under content and contact risks. The most common risks named in the EU Kids Online report (Livingstone and Haddon 2009) refer to these two categories as well: chatting with online contacts not met before, cyber bullying, pornography, sexting, texting, websites with harmful user-generated content (e.g., hate, pro-anorexia, drug taking or suicide), personal data abuse, and excessive Internet use. In line with previous research, the term online risk behavior used in this study refers to online behaviors that are both content and contact risk related.

Although most surveys tend to focus on adolescents, research on primary school Internet users are needed since children go online at ever younger ages. The average age of first Internet use is 7 years in Denmark and Sweden and 8 years in several other Northern European countries (Livingstone and Haddon 2009). Although the age at which children are going online is decreasing, teacher’s engagement with children’s Internet use is least among 9–10 year olds compared to older children (Livingstone and Haddon 2009). Supporting children in their Internet use at a younger age is stressed in the Byron review (2008). Here, it is stated that young children rely on others in making their choices since they are still immature in self-regulation, and their ability to inhibit and control impulses is still low. Byron (2008) makes a useful analogy between young children on the Internet and young children in public swimming pools. Before we let children go to swim, we first teach them how to swim; there are lifeguards who watch them, and there are swimming aids available for the younger swimmers (Byron 2008).

Raising awareness in children about dangers on the Internet is important before they move into adolescence, since one of the most prominent changes in early adolescence is the shift from compliance and commitment to the parents toward peer orientation (Fuligni and Eccles 1993). Early adolescents tend to assert their autonomy and start to individuate themselves from their parents. In this developmental period, adolescents start exploring boundaries originally set for them by their parents, thereby making them more susceptible for asserting risk behavior (Steinberg 2004). Additionally, boys tend to be more risk taking than girls (Morrongiello and Rennie 1998). Though risk taking is a developmental imperative in early adolescence and mostly inevitable, it seems important to intervene during, or right before, the transition toward early adolescence. This is the same phase in which they tend to start exploring the Internet by themselves. The current study will therefore focus on children in this developmentally important phase.

The first policy alternative for the protection of minors in cyberspace would be parental control and supervision, logically, because the ability of parents to restrict the amount of time that children can spend on the Internet. Parents do seem to talk about the risks on the Internet with their children (70 %; Livingstone and Haddon 2009) and parental monitoring seem to diminish online harassment (Khurana et al. 2014). On the contrary, most children who have encountered an online risk did not tell their parents about this (Livingstone and Haddon 2009). Also, when it comes to safety on the Internet, parents came to rely on what Livingstone (2002, p. 250) calls “the European context of strict broadcasting regulation for protection of minors”. It is thus not surprising that the majority of parents state that they want more information from public organizations such as schools and local authorities (Livingstone and Haddon 2009). Relying on parents solely to promote safer Internet use in children would be problematic since not all parents are equally capable to carry out this task because of the current generation gap in Internet skills between them and their children (Livingstone and Bober 2006).

There thus seems to be an increasing demand and responsibility for primary schools to develop educational and preventive programs concerning risky behavior on the Internet. This seems even more evident when considering that school work is the main online activity reported by children (Livingstone and Haddon 2009). The idea of giving the responsibility of informing about the Internet to schools is in accordance with the policy recommendations made in the EU Kids Online report (EUKO; Lobe et al. 2011). In this report, it is stated that schools have the resources to reach all children and should therefore attempt to inform all children about proper Internet use. More mediation of teachers in the young group of children could possibly reduce the risks that young children may encounter when on the Internet.

Although the online safety of children seems like a widely discussed topic, there is a scarcity of empirical information concerning ways to intervene in their online behavior. An overview of qualitative and quantitative studies concerning the Internet and other media in the Netherlands reveals that the majority of the teachers in primary school feels that there is not enough educational material available concerning this topic (Van Grinsven et al. 2011). Even more evident, to our best knowledge there are no empirically tested interventions to decrease online risk behavior in primary school children. Specifically focusing on risky behavior on the Internet, several awareness campaigns were launched in the last decade (Valcke et al. 2007). Awareness campaigns are widely used by local governments and the European Union to promote positive behavior in children. However as mentioned before, most studies concerning safe Internet use campaigns specifically are descriptive in nature and there tends to be a lack of evaluative research concerning the effect of such interventions (for an overview see Valcke et al. 2007).

Empirical studies on the effect of awareness raising interventions on the offline behavior of children can be found on topics such as physical aggression among peers. An example is the VERB campaign that is embedded in scientific literature (Huhman et al. 2010). VERB was a health marketing campaign from 2001 to 2006, targeting 9–13 year olds to be physically active every day. The awareness campaign entailed television advertising, school directed promotions, and community based activities. Children who were aware of the campaign were more likely to be physically active compared to the children who noted that they did not hear from the campaign. Another example of a successful awareness raising campaign to alter behavior was a school-based anti-violence intervention for middle schools. The intervention included use of media and classroom presentations showing other ways to solve a conflict (Swaim and Kelly 2008). The intervention had a positive effect on both the cognitive and behavioral aspects of the behavior of the youngsters. These outcomes show that awareness raising interventions can have a positive effect on children’s behavior, which might also be effective to alter their online behavior.

The idea that increasing awareness can alter behavior is backed up by research and traditional models of behavioral change. The Theory of Planned Behavior by Ajzen (1991) suggests that behavior is dependent on one’s intention to perform the behavior. Behavioral intention, in its turn, is influenced by the attitude of a person with certain beliefs and values about the outcome of the behavior, subjective norms based on normative beliefs, and his perceived behavioral control that depends on control beliefs. This behavioral theory suggests that it may be important to present information to change attitudes toward the wanted behavior. The importance of information provision is also argued in the trans-theoretical stages of the change model by Prochaska et al. (1998). This model suggests that people follow several steps toward behavioral change. It is essential to have a planned intervention to travel through the first steps of the model toward behavioral change. One way is to increase awareness of the risk, for example by educational materials and informing about the behavior (Prochaska et al. 1998). Both models stress the importance of informing and awareness raising interventions to promote behavioral change, in this case to reduce online risk behavior.

## Current Study

In line with the tradition of governmental and European activities to promote health behavior by raising awareness, the current study will examine whether a school based intervention is effective in raising online risk awareness, and thereby online risk behavior. We refer to online risk awareness by examining children’s awareness of both contact and content risk, categories named by De Moor et al. (2008). The intervention will aim to increase online risk awareness in children in the transition toward and in early adolescence (6th–8th grade; age 8–14) since children in this age range explore the Internet by themselves and tend to assert their autonomy rather than obey the rules that are set for them. It thus seems important to intervene before or during this transition to adolescence in order to teach children how to behave safely online. Classroom interventions including a presentation on online risks will be provided for half of the children participating in this study in contrast to children in the control group who will receive a neutral based presentation concerning the Internet. The effect will be examined at two time points, directly after the intervention and 4 months after.

Based on the discussed literature, we expect that a school-based educational intervention will raise the online risk awareness of children, and although diminishing, this effect is expected to still be visible 4 months after. The intervention is also expected to reduce online risk behavior in the months after the intervention compared to children who did not receive the intervention. It is expected that awareness will act as a moderator, children with more awareness are expected to show less online risk behavior when they had the intervention compared to children who are less aware of the risks online and had the intervention. Furthermore, individual differences will be assessed. Literature suggests that older children who are moving toward early adolescence tend to be more risk taking than their younger peers (Steinberg 2004), in which boys are more likely to assert risk behavior compared to girls (Morrongiello and Rennie 1998). To the best of our knowledge, this is the first study to compare two conditions and its effect on online risk awareness and behavior of early adolescents. Therefore, our research will hopefully provide a valuable starting point to expand the literature on the effectiveness of online risk-related interventions for primary school children.

## Methods

### Procedure

This study was conducted in the five provinces of Flanders (Limburg, Antwerp, East Flanders, West Flanders, and Flemish Brabant) in Belgium. Fifteen Flemish primary schools were selected from the three official Flemish Educational Networks, namely Community education, Subsidized publicly run education (e.g., schools of municipals) and Subsidized privately run education (e.g., Catholic education). In total 22 classes from 15 schools were selected to participate in the intervention study. Per province, classes were randomly assigned to the intervention or control group. Nine classes (T1 n = 355; T2 n = 360) were assigned to the control condition and did not receive the intervention, 13 classes (T1 n = 457; T2 n = 459) did receive the intervention. The same classes participated at both measurements Time 1 and Time 2.

**Fig. 1:**
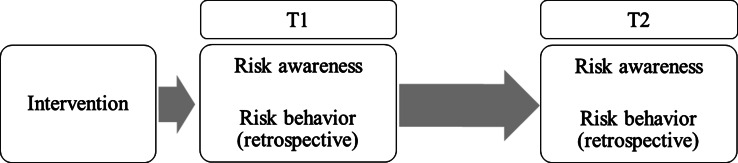
Timeline and variables measured at both Time 1 and Time 2

Data were collected from November to December 2012 (Time 1), and again in May 2013 (Time 2). Prior to the first measurement, a 10-min intervention was carried out per class by a research associate. Immediately after the intervention, self-report questionnaires were assessed anonymously, in class, and in the presence of a researcher. The second measurement was assessed 4 months later in the same school year. The same classes were asked to complete a questionnaire identical to that of the first measurement 4 months earlier. Note that risk awareness and risk behavior were measured at both times, but that risk behavior was measured retrospectively, asking about their behavior in the past 6 months. So at Time 1 referring to a period before the intervention, see also Fig. 1.

The intervention was a presentation by a research associate with the emphasis on risk that children can encounter on the Internet. Five topics concerning online risk behavior were addressed: textual contact over the Internet (e.g., chatting, grooming, bullying), audio-visual contact (e.g., bullying, extortion and strangers), social network services, online games, and offline meetings with people met online. For a detailed description of the intervention presentation, see “Appendix 1”. The control group received a neutral informative presentation by a research associate about two Internet applications (Wikipedia and OpenStreetMap), without any emphasis on online risks (See “Appendix 2”). Both presentations took approximately 10 min and were presented in their own classroom.

### Sample

The final sample at Time 1 consisted of 812 children from fourth (*n* = 350) and sixth (*n* = 462) grade. This sample consists of 51.2 % girls (*n* = 416) and 48.2 % boys (*n* = 391); for five children, gender information was missing. The majority of children (47.8 %) were born in 2001 (*n* = 388) and 2003 (37.6 %, *n* = 305). The average age (an approximation based on year of birth) in fourth graders was *M* = 9.10, *SD* = .37 and in sixth graders *M* = 11.13, *SD* = .39 the youngest were 8 years old at Time 1 and the oldest fourteen.

The final sample at Time 2 consisted of 819 children from fourth (*n* = 351) and sixth (*n* = 468) grade. The sample consists of 51.3 % girls (*n* = 420) and 48.7 % boys (*n* = 399). The majority of children (48.5 %) were born in 2001 (*n* = 397) and 2003 (37.7 %, *n* = 309). The average age (an approximation based on year of birth) in fourth graders was *M* = 10.08, *SD* = .37 and in sixth graders *M* = 12.13, *SD* = .39 the youngest were 8 years old at Time two and the oldest fourteen.

Due to the request of almost all participating schools to guarantee total anonymity of the teachers, it was impossible to match the scores of the children over Time 1 and Time 2 Therefore analysis in this study are examined for the mean scores of both measurements separately.

### Measures

#### Online Risk Behavior

The dependent variable Online Risk behavior was measured by 15 questions, all referring to online behavior carried out in the last 6 months. This scale covered several online behaviors which are considered as risky. The following topics of risk behavior were included: talking, gaming, webcamming, meeting, or chatting with a stranger met online, getting to know someone online, providing the own home address or phone number on a public profile, having an e-mail address parents do not know of, using the Internet without parents knowing, pretending to be older or pretending to be someone else. An example question is: “Have you got to know someone online you didn’t meet in real life?” Answer categories were dichotomous with 0 = “never done this” and 1 = “I did this at least once”. A mean score was calculated for these questions thus ranging from zero to one, with a higher score indicating more online risk behavior. The reliability of the scale was good with Cronbach´s alpha = .77 at Time 1 and alpha = .81 at Time 2.

#### Online Risk Awareness

The dependent variable Online Risk Awareness was measured with nine statements concerning potentially risky activities on the Internet. Children were asked to answer to each statement on a scale from 1 to 5 with 1 = very safe, to 5 = very unsafe. A mean score was calculated for the nine statements thus scores ranged from one to five, with five indicating more online risk awareness. The following subjects were covered by the nine statements: chatting with strangers, using non- concealing chat alias, sharing cellphone number with strangers, opening an unknown e-mail attachment, webcamming with strangers, using public social network sites, clicking on fraudulent web links and pretending to be older. An example statement is: “Sarah is a 10-year old girl. After school, she regularly chats on a website with girls and boys she does not know. That way, she gets to know new friends”. The reliability of the scale was good (Cronbach’s alpha = .79 at both Time 1 and Time 2).

#### Condition

The independent variable was whether or not the child received the intervention at Time 1.

#### Covariates

Both grade and gender were entered as covariates in the analyses since both were expected to be related to the outcome variables in this study, namely online risk awareness and online risk behavior. Literature suggests that boys and older children tend to be more risk taking than their female peers and younger children (see for example Morrongiello and Rennie 1998; Steinberg 2004). Both were dummy coded: grade with 0 = 4th grade and 1 = 6th grade and gender with 0 = girl and 1 = boy.

### Statistical Analysis

All data were analyzed using SPSS 22 for Windows and a *p* value of .05 was used to determine the significance of the effects. Due to the request of almost all participating schools to guarantee total anonymity of the pupils, no individual numbers or scores could be matched over time, repeated measures were therefore impossible to calculate. Therefore comparisons of the mean scores were made with the use of analysis of covariance.

First, correlations between the covariates, independent and dependent variables were calculated to explore the data. To further analyze whether the condition (control/intervention) was associated with the two outcome variables (i.e., online risk behavior and online risk awareness), analyses of covariance were conducted. Both gender and grade were used as control variables in all analyses. To examine the link between online risk awareness and online risk behavior stepwise multiple hierarchical regression analysis were computed. An interaction variable was entered to examine the expected moderating role of online risk awareness on the link between the intervention and online risk behavior.

## Results

### Correlations

#### Time 1

Online risk awareness was positively correlated with participation in the intervention group. Being in a higher grade was associated with more online risk behavior and boys were less aware of online risks than girls. Online risk behavior correlated negatively with online risk awareness and was not correlated with the intervention at Time 1. For all correlations, see Table 1.

**Table 1:** Correlations of the covariates (gender/grade), independent (condition), and dependent variables (online risk awareness/behavior) for Time 1

	Condition	Awareness	Risk behavior	Grade	Gender
Condition	–	–	–	–	–
Awareness	.322^**^	–	–	–	–
Risk behavior	.036	−.272^**^	–	–	–
Grade	−.025	−.067	.163^**^	–	–
Gender	−.035	−.134^**^	.118^*^	.033	–

Condition: 1 = intervention, Grade: 1 = 6th grade, Gender: 1 = boy

* *p* < .05; ** *p* < .001

#### Time 2

All correlations at Time 2, except the correlation between online risk behavior and the condition, resembled that of Time 1 as described above. The intervention was associated with more online risk behavior. For all correlations, see Table 2.

**Table 2:** Correlations of the covariates (gender/grade), independent (condition), and dependent variables (online risk awareness/behavior) for Time 2

	Condition	Awareness	Risk behavior	Grade	Gender
Condition	–	–	–	–	–
Awareness	.105^**^	–	–	–	–
Risk behavior	.082*	−.422^**^	–	–	–
Grade	−.031	−.022	.221^**^	–	–
Gender	−.018	−.134^**^	.139^**^	.039	–

Condition: 1 = intervention, Grade: 1 = 6th grade, Gender: 1 = boy

* *p* < .05; ** *p* < .001

### Online Risk Awareness and the Intervention

#### Time 1

A one-way ANCOVA was conducted to examine whether there was a statistically significant difference in online risk awareness between children who did or did not receive the intervention directly after the intervention took place. Grade and gender were hereby entered as covariates. Gender was correlated with online risk awareness [*F*(1, 803) = 13.31, *p* < .001, *r* = −.14]. Boys were less likely to be aware of online risks (*M* = 3.88, *SD* = .62) than girls were (*M* = 4.05, *SD* = .58). Grade was not correlated with online risk awareness. There was a significant effect of the intervention on online risk awareness after controlling for gender and grade [*F*(1, 803) = 90.92, *p* < .001, *partial* η^2^ = .10]. Participation in the intervention group was related to more online risk awareness (*M* = 4.14, *SD* = .55) while participation in the control group was associated with less online risk awareness (*M* = 3.75, *SD* = .60).

#### Time 2

Another one-way ANCOVA was conducted to examine whether there was still a statistically significant difference in online risk awareness 4 months after the intervention between children who did or did not receive the intervention. Gender was significantly correlated with online risk awareness. Boys were less likely to be aware of online risks (*M* = 3.69, *SD* = .66) than girls were [*M* = 3.86, *SD* = .62; *F*(1, 813) = 14.47, *p* < .001, *r* = −.13]. Grade did not significantly explain online risk awareness. As at Time 1 there was a significant effect of the intervention on online risk awareness after controlling for gender and grade [*F*(1, 813) = 8.72, *p* < .005, *partial* η^2^ = .01]. Although the effect is smaller than at Time 1, participation in the intervention group was still related to more online risk awareness (*M* = 3.84, *SD* = .62) compared to children in the control group (*M* = 3.70, *SD* = .66) 4 months after the intervention.

### Online Risk Behavior and the Intervention

#### Time 1

Since the measurement of online risk behavior is retrospective, this variable covers the 6 months before Time 1, so before the intervention took place. An analysis of covariance was conducted to confirm that no differences existed between the children who got the intervention and the children who had not.

Again, gender and grade were entered as covariates. Both gender and grade were related to online risk behavior. Boys were more likely to show online risk behavior (*M* = .233, *SD* = .197) than girls were [*M* = .187, *SD* = .171; *F*(1, 800) = 9.77, *p* < .005, *r* = .10]. Also children in the sixth grade were more likely to report online risk behaviors (*M* = .237, *SD* = .189) compared to children in the fourth grade [*M* = .176, *SD* = .175; (*F*(1, 800) = 24.70, *p* < .001, *r* = .17]. After controlling for gender and grade no significant difference was found for receiving the intervention and online risk behavior at Time 1.

#### Time 2

Another analysis of covariance was conducted to examine whether there was an association between the intervention and online risk behavior at Time 2. Again, gender and grade were entered as covariates. Both gender and grade were related to online risk behavior. Again, boys were more likely to engage in online risk behavior (*M* = .279, *SD* = .214) than girls were [*M* = .223, *SD* = .187; *F*(1, 812) = 13.66, *p* < .001, r = .14]. Also children in the sixth grade were more likely to report online risk behaviors (*M* = .288, *SD* = .120) compared to children in the fourth grade [*M* = .198, *SD* = .195; *F*(1, 812) = 49.53, *p* < .001, *r* = .27]. After controlling for gender and grade, a significant effect of the intervention on online risk behavior was found [*F*(1, 812) = 8.38, *p* < .005, *partial* η^2^ = .01]. Participation in the intervention group was related to more online risk behavior (*M* = 3.84, *SD* = .62) compared to children in the control group (*M* = 3.70, *SD* = .66).

### Online Risk Awareness and Online Risk Behavior

With two hierarchical multiple regression analysis it was examined whether online risk awareness could predict the amount of online risk behavior.

#### Time 1

In the hierarchical linear regression analyses predicting online risk behavior, the covariates gender and grade entered in step 1 explained 4.2 % of the variance at Time 1. Both grade and gender significantly predicted online risk behavior, with boys and being in a higher grade reporting more online risk behavior compared to girls and being in a lower grade. In the second step, awareness added 6.4 % to the variance. The predictor and the covariates explained 10.6 % of the variance in the online risk behavior scale, *F*(3, 800) = 31.46, *p* < .001. Online risk awareness significantly predicted online risk behavior (*B* = −.26, *t* (806) = −7.54, *p* < .001). As expected, more online risk awareness was associated with less online risk behavior.

#### Time 2

In the hierarchical linear regression analyses predicting online risk behavior, the covariates gender and grade entered in step 1 explained 7.1 % of the variance at Time 2. Both grade and gender significantly predicted online risk behavior, with boys and being in a higher grade reporting more online risk behavior compared to girls and being in a lower grade. In the second step, online risk awareness added 14.9 % to the variance. The predictor and the covariates explained 22.0 % of the variance in the online risk behavior scale, *F*(3, 811) = 76.31, *p* < .001. As at Time 1 online risk awareness significantly predicted online risk behavior (*B* = −.39, *t* (814) = −12.44, *p* < .001). Higher scores on online risk awareness were associated with asserting less online risk behavior.

This effect remained when intervention was entered in the last step to control for the effect of the intervention. The intervention added 2.0 % to the variance (*p* < .001). In total, the predictor and the covariates explained 24.0 % of the variance in the online risk behavior scale, *F*(4, 810) = 64.12, *p* < .001. As seen before in the analysis of covariance the intervention significantly predicted online risk behavior (*B* = .14, *t* (814) = 4.66, *p* ≤ .001). Awareness thus still significantly predicted online risk behavior when intervention was entered into the model (*B* = −.41, *t* (814) = −13.03, *p* < .001).

### Moderating Role of Awareness

To test whether awareness moderates the effect of the association between the intervention and online risk behavior an interaction variable (online risk awareness × intervention) was computed and entered in the last step of the hierarchical regression model. The moderating role of online risk awareness was examined for Time 2 since online risk behavior at Time 1 refers to a time frame before the intervention and can thus not be predicted by the intervention. The steps previous to adding the interaction variable are the same as described in the section above. Covariates grade and gender were added in the first step of the analysis and awareness and intervention in the second step.

In the third step, the interaction variable was added to examine a possible moderation effect. The predictors online risk awareness, intervention, the interaction variable and the covariates explained 24.0 % of the variance in the online risk behavior scale, *F*(5, 809) = 51.95, *p* < .001. However this could not be assigned to adding of the interaction variable since the model did not change significantly (*∆R2* = .003, *p* = .10, *ns*). The interaction variable online risk awareness × intervention did not predict online risk behavior (*B* = −.01, *t* (814) = −1.65, *p* = .10, *ns*).

## Discussion

Despite several policies to reduce the risks that children encounter on the Internet, there is only a limited amount of studies focusing on the impact of school-based Internet safety interventions (Valcke et al. 2007). The current study revealed that a school-based intervention can have a positive effect on the online risk awareness directly after the intervention, and although diminishing, this effect on online risk awareness was still present 4 months after the intervention. Surprisingly, children who had the intervention were more likely to report online risk behavior 4 months after the intervention and awareness did not moderate this effect. Still, more online risk awareness in general was associated with less risk behavior online. Individual differences in both age and gender were observed. Confirming our expectations, girls were more likely to be aware of online risks than boys were. Additionally, boys and children in a higher grade were more likely to engage in online risk behavior.

As expected, the intervention had a positive effect on online risk awareness. Although the association between the intervention and more online risk awareness was more evident directly after the intervention, it was still noticeable 4 months after the intervention. It thus seems that a relatively short intervention was successful in raising the awareness of children about risks on the Internet. This is in line with the two models of behavioral change by Ajzen (1991) and Prochaska et al. (1998), which propose that awareness should be increased by presenting information and educational materials to inform about the wanted behavior. Additionally, the findings confirm previous literature concerning evidence based school interventions, which show that children are more aware of the online risks and desired online behavior after they received an information based intervention (Huhman et al. 2010; Swaim and Kelly 2008). The intervention thus achieved a relative increase in awareness up to 4 months after the intervention despite the relatively short duration and moderate intensity of the intervention.

However, the fact that there was an increase in awareness in the children who received the intervention did not necessarily mean that this group showed less online risk behavior in the months after the intervention. Compared to the control group, children who received the intervention showed no decrease in online risk behavior. It is surprising that the children who received the intervention even engaged in relatively more online risk behavior 4 months after compared to children who did not receive the intervention. The lack of an intervention effect, or even the occurrence of the opposite effect, on online risk behavior is against our expectations derived from the behavioral change theories by Prochaska et al. (1998) and Ajzen (1991) that propose that an increase in awareness will change the behavior as a consequence. When examining the direct effect of online risk awareness and behavior, it was revealed that a higher online risk awareness was indeed related to less online risk behavior as expected from both models of behavioral change (Prochaska et al. 1998; Ajzen 1991). Additionally, when it was examined whether awareness affected the association between the intervention and a higher amount of online risk behavior, this did not seem to be true. This finding indicated something different than just an increase of awareness is happening within the group of children that received the intervention.

One possible explanation for the fact that children in the intervention group had more online risk awareness compared to children who did not receive the intervention but also reported more online risk behavior could be explained by a difference in reporting. In other words, these children might have also become more aware of their own behavior in the months after the intervention, which is reflected in the reporting of their behavior. This could have resulted in a better memory of their violations of safe Internet use and report more online risk behavior as a consequence. This idea aligns with studies on obese adults. Adults who were conscious of their own eating behavior and what they should change about it, focused more on their eating behavior and were better able to remember and report their failures to commit to the diet (Castellanos et al. 2009). This focus on the unwanted behavior, then, does not necessarily mean that they also asserted more online risk behavior compared to the control group. The incidence of actual online risk behavior might be difficult to capture without obtaining any information to verify the information reported by the children. Future research could use a multi perspective by adding the view of third parties, such as school or parents, or track computer records to get a more accurate view of online risk behavior to overcome a possible difference in attention toward the norm behavior in the intervention group.

Furthermore, the results of the current study revealed that it is important to take individual differences into account when focusing on online risk behavior. Boys were more likely to show online risk behavior compared to girls. This is in line with the literature that suggests that boys tend to be more risk taking than their female peers (Morrongiello and Rennie 1998). According to a Portuguese study in 8–17 year olds, most boys can be identified as intensive Internet users without parental mediation, whereas most girls are classified as moderate users with parental mediation (de Almeida et al. 2010). More intensive Internet use together with more general risk taking in boys makes it plausible that boys stand a higher chance to (un)consciously engage in online risk behavior. The current study also showed that girls were already more aware of online risks compared to boys, indicating that interventions to increase awareness should consider giving special attention to boys. This is supported by a study by Segers and Verhoeven (2009), which revealed that boys were more likely to benefit from a sheltered Internet environment than girls did since boys tend to browse more on the Internet.

Additionally, differences in online risk behavior, but not online risk awareness, were found with regard to age. This is in line with research by Steinberg that showed that children in early adolescence did not differ in risk awareness compared to younger children (Steinberg and Cauffman 1996). Yet, older children were more likely to show risk behavior, which is an imperative in this shift toward early adolescence (Morrongiello and Rennie 1998; Steinberg 2004). This can be explained by several developmental changes associated with early adolescence. Adolescents develop a stronger system of novelty and reward seeking which makes them more likely to engage in risky behaviors. This is accompanied by relatively slow development of self-regulatory capabilities that makes adolescents less likely to move away from risks. Since novelty and award seeking starts in early adolescence, and self-regulation does not fully develops until late adolescence, this makes adolescents more susceptible for asserting risk behavior despite the fact that they are aware of risks (Steinberg 2004). As stressed before, developmentally, it might be important to intervene at an early age. This makes it possible to assert influence on their behavior online instead of intervening in the period of adolescence itself in which children tend to assert their autonomy and change focus from their caregivers toward peers (e.g., Fuligni and Eccles 1993; Pasquier 2001; Byron 2008; Luna and Finkelhor 1998).

Despite careful consideration of the research model, it is important to note some limitations of the current study. At first, due to strict anonymity regulations of the participating primary schools, using a prospective design was not possible. Therefore, the design was cross-sectional in nature, making it difficult to draw any causal inference. Although no causal inferences can be made, we believe that this study provides a valuable exploration of interventions with regard to online behavior in young children. Future research should focus on longitudinal studies to further explore the effect of a school-based intervention to reduce both online risk awareness and behavior. Questionnaires were carefully developed and tested in their understandability for the purpose of this study. Although the currently used questionnaires were not validated before, questionnaires were tested on their reliability, which were sufficient for both scales. It is important that future research in the field consider using existing questionnaires to see whether the reliability of the current questionnaire will give the same reliability outcomes in other studies. The current study shows that it is important to assess individual differences such as gender and age; however, cultural differences were not examined in the current study due to a lack of cultural varieties in the sample. Future studies are encouraged to assess the influence of cultural differences since previous study do reveal an effect of ethnic origin on online risk behavior such as engagement in cyber bullying (e.g., Shapka and Law 2013).

The current study has a number of strengths that are important to note. First is the large sample size of the current study. This allowed us to explore a relatively unexplored field of research with high practical relevance. By comparing group outcomes of the intervention versus the non-intervention group valuable first information was provided concerning the effect of a school-based intervention on not just awareness but also how this translates to actual behavior. The current study will hopefully provide a valuable starting point for further research concerning this topic. Furthermore, the current study carries implications for future intervention research concerning the effectiveness of awareness raising interventions and its influence on actual behavior. Additional research concerning the link between online risk awareness and online risk behavior is needed.

Most important is the practical relevance of the current study for educational institutes and policy makers. Since most teachers and parents stress that they would like to have more information concerning the activities of children on the Internet, and most importantly how to guide them, the current research started to fill a gap in this knowledge field by examining the effect of a school based intervention (Van Grinsven et al. 2011; Livingstone and Haddon 2009). To our best knowledge there were no empirically tested interventions to decrease online risk behavior in primary school children. Although we acknowledge that parents play an important role in educating their children, we feel that schools have an increasing responsibility to take the lead with starting to educate children about the Internet. This idea is in accordance with the policy recommendations made in the EU Kids Online report in which it has been stated that schools have the resources to reach all children and should, therefore, attempt to inform all children about proper Internet use (EUKO; Lobe et al. 2011). Although general advice to caregivers should be given cautiously, we hopefully provided a first guideline by proposing a possible intervention that can be adjusted and improved when more research on this topic will follow. This would allow teachers to better intervene in the Internet behavior of primary school to reduce the risks that young children may encounter when on the Internet.

Several suggestions can be made for future adaptions on the intervention program. A valuable overview of ways to promote actual behavioral change was made by Luna and Finkelhor (1998) by analyzing successful intervention programs. They suggest that a successful intervention program should repeatedly invite people to focus on skill development, include interactive instructional strategies, and consider individual differences (Luna and Finkelhor 1998). For a future study it might, therefore, be important to invest in a longer term intervention embedded in the education program, actually teaching children technical skills to protect from harm when online. In this context, the successful school-based program Net-Detectives to reduce online risk behavior (Wishart et al. 2007) is worth mentioning. Through computer-based role play, children became detectives who investigated misuse of school computers and practiced their computer skills with peers. The success of this study reveals that an intervention that includes a more active role of children as well as education about technical skills can achieve safer Internet behavior (Wishart et al. 2007). However, future research should also investigate whether this effect in childhood persists in adolescence. As argued by Steinberg (2004), children in this age period do not differ in their awareness of what is risky, but this does not diminish their actual risk behavior. As stressed before, this could be attributed to the increase of peer pressure and novelty seeking, but a decrease in self-regulation (Steinberg 2004). It, thus, seems important for future studies to investigate whether the effect of an early intervention holds through adolescence.

## Conclusion

The current study sheds light on a relatively unexplored research field concerning the effect of a school-based intervention on both online risk awareness and behavior. Since most research in this field is descriptive in nature, the current research provides a first study on the effects of a school-based intervention on online risk awareness and online risk behavior in children in the transition toward early adolescence. Within the limitations of the design, the current study reveals that a relatively short school-based intervention positively affects online risk awareness up to 4 months after the intervention. An increase in awareness was associated with lower amounts of reported online risk behavior. Despite a higher amount of online risk awareness, reporting of online risk behavior in the intervention group was higher compared to the group of children who did not receive the intervention that was not moderated by awareness. It is proposed that this might be a difference caused by an attentional bias, children in the intervention group were more likely to focus on their own behavior and, therefore, report more as a consequence. Although it is important to verify current outcomes through future studies, outcomes of the current study can have great educational implications. Additionally, individual differences were exposed. As expected, girls were more likely to be aware of online risks and asserted less online risk behavior compared to boys. Furthermore, results were in line with the expectation that risk taking becomes more of an imperative in adolescence. Specifically, the current study confirms that older children did not differ from younger children in their online risk awareness, yet they did show more online risk behavior. The Internet might have a lot of positive aspects to offer for children, but to benefit from those, it is important to examine how we can decrease the risk that they encounter online. To refer back to the Byron review (2008), Internet is like swimming, it is a lot of fun but it is important to learn how to swim first.

## Appendix 1

In this Appendix it is described how the intervention was conducted and what it included. Note that all slides were built up using very easy wordings and graphics. All sides are available via Dataverse.

Power Point Sheets of the Intervention, will be made publicly available via Dataverse as stated in the article.

Powerpoint Sheets of the Control Group will be made publicly available via Dataverse as stated in the article.

### The Title

The intervention presentation was titled “Op de computer en het Internet zitten kan soms gevaarlijk zijn” which can be translated as “Using computers and the Internet can possibly be dangerous”. When the title slide was shown, the children were explained what was meant with “can possibly be dangerous”. It was explained that using a computer is not dangerous in definition and they shouldn’t be afraid of doing so but that it depends highly on how you behave and how aware you are of possible risks and ways to avoid them or cope with them.

### First Topic: Textual Contact

This first topic focusses broadly on risks associated with textual contact with others over the Internet. The wordings and icons on the slides of this topic refer to chatting and e-mailing and the children are given examples orally to make clear that this embraces all kinds of messaging on different platforms. The first risk of this activity that was discussed is the fact that you cannot be sure of the true identity of your chat-partner if you haven’t met him or her before. A first slide depicts a chat/e-mail partner who says “Hi, I’m Laura, I’m 10 years old” as a girl silhouette with a question mark. In the next slide, this girl is exchanged for an evil looking man. The slide now also features 2 “danger”-signs: one with an exclamation mark and the word “strangers” and one with a question mark and the word “trust”. The message given to the children is that, because you cannot know for sure the identity of strangers you have textual conversations with, one should be very careful not to trust strangers too much or too quickly. They are told be very cautious with passing on personal data to conversational partners (because of the risk of for example grooming, identity theft, etc.).

A next slide points to the risks of being bullied in a textual conversation and getting computer virus infections; 2 danger signs warn for “bullies” and “viruses”. The children are given the message that bullying might happen not only on the playground but using on textual Internet media too with some examples. It is also explained how a computer can be contaminated with a computer virus (e.g. by clicking on fraudulent links or receiving data transfers). In the following slide, this is overlaid with a big danger-sign and the word “undesirable”. Now the children are given more examples of possible risks that can occur via textual media: e.g. unwanted solicitation, grooming…).

### Second Topic: Audiovisual Contact

The second part of the presentation starts off with a slide showing a webcam and a headset icon and the words “webcam and talking”. This part embraces all kinds of audiovisual contact: where contacts can see or hear the contact partner. The following slide shows the webcam and headset icon and a silhouette with a question mark on it. The children are now told that even they can hear and/or see their contact, they can still not be sure about the real identity of the conversational partner: they are taught that voice or even video pre-recordings could be used by malign people to adopt a false identity. In a next slide, a picture of a camera operator and a “recording” picture are added while the children are explained that everything they say or do on webcam or during an audiovisual call could be recorded by the contact and used against them (e.g. for bullying, extortion …) and that once something is posted online somewhere it can be very difficult to remove. The last slide on this topic adds four danger signs with the words “bullies”, “undesirable”, “strangers” and “trust”; several examples of risks are given to the children with inter alia repetitions of the advices already given with the former topic.

### Third Topic: Social Network Services

The first slide on this topic shows a number of logos of well-known social network services. The children are asked if they recognize these logos and to give more examples they know of. The slide also depicts a silhouette of a boy with a question mark on it. Again the children are taught that, also using these media, once cannot be sure of the real identity of the people they have contact with. On the next slide, the boy silhouette is replaced with the “evil man” picture that was already used before. The “virus” picture (that was used in a former topic) reappears as well as 5 danger sign with the words “bullying”, “undesirable”, “strangers”, “trust” and “viruses”. The children are repeatedly warned about the different risks of bullying, computer virus infections and risks associated with trusting strangers too much or too quickly.

### Fourth Topic: Online Games and Contests

This topic is started with a slide depicting several images luring people to enroll in online games and contests. The children are asked if they recognize these and what they are. In a next slide, these pictures are “stained” with the “virus” image used before and a danger sign with the word “viruses”. The children are taught that lots of these games or contests are fraudulent and clicking on these pictures might cause risks like for instance a computer virus infection. The children are told to be vigilant and skeptical about these contests and to be aware that they might be made by people with bad intentions. A next slide adds 4 more danger signs with the words “trust”, “strangers”, “undesirable” and one with a euro-sign (€). The message to the children is to be very cautious with clicking on links and images that might try to lure them into downloading malicious software or filling in personal data (e.g. filling in a cell phone number or e-mail address and unwillingly being subscribed to a costly SMS service or spamming plague).

### Fifth Topic: Offline Meetings with People Met Online

The last part of the presentation is about setting up a meeting with a stranger met online. After the title-slide, the image of 2 people handshaking is overlaid with a keyboard picture and a speech bubble with the text “Hello, I’m Laura, I’m 10 years old…”, the same salutation used in the first slide of the topic on textual contact. Using the same text should ring a bell in the children’s mind on what to expect. In the next slide, one of the handshaking figures is overlaid with 4 silhouettes: one of a girl, a boy, a woman and a man. The message being: you cannot know for sure who you are going to meet in real life when you set up a meeting with an online contact. In a next slide these silhouettes are replaced with the (by know well-known) image of the “evil man”. The last slide adds the already familiar danger signs with the words “trust”, “strangers” and “undesirable”. The children are told about some of the risks they might encounter when setting up a meeting with a malign contact and asked to share their thoughts.

## Appendix 2

In this section the presentation that the control group received is described. Again, the slides were built up using very easy wordings and graphics. All sides are available via Dataverse.

### Title

The title was Wikipedia and OpenStreetMap which is meant as a neutral title to introduce the topics of the presentation.

### Wikipedia

Slides were all used to explain the use of Wikipedia as an online Encyclopedia. Slides were neutral and did not include any signaling symbols in comparison to the slides used in the intervention group. The slides and the accompanied presentation of the research associate explained that Wikipedia is an online version of the Encyclopedia books that people used before. Possibilities were explained to search for a topic and that everybody is able to contribute to the Online Encyclopedia.

### OpenStreetMap

Slides in the second part of the presentation were all used to explain the use of OpenStreetMap. Slides were neutral and did not include any signaling symbols in comparison to the slides used in the intervention group. The slides and the accompanied presentation of the research associate explained that OpenStreetMap is an online tool to develop, and look for maps. It was explained that everyone is able to contribute and that you can get different kind of maps free of charge.

## References

[CR1] Ajzen I (1991). The theory of planned behavior. Organizational Behavior and Human Decision Processes.

[CR3] Byron, T. (2008). Safer children in a digital world: The report of the Byron Review: Be safe, be aware, have fun.

[CR4] Castellanos EH, Charboneau E, Dietrich MS, Park S, Bradley BP, Mogg K, Cowan RL (2009). Obese adults have visual attention bias for food cue images: Evidence for altered reward system function. International Journal of Obesity.

[CR5] De Almeida, A. N., Delicado, A., & De Almeida Alves, N. (2010). Children and the Internet in Portugal: A diversified portrait. *Society, Culture and Technology at the Dawn of the 21st Century*, 143.

[CR6] De Moor, S., Dock, M., Gallez, S., Lenaerts, S., Scholler, C., & Vleugels, C. (2008). Teens and ICT: Risks and opportunities. Retrieved July 6, 2010 from. http://www.belspo.be/belspo/fedra/TA/synTA08_nl.pdf.

[CR7] Fuligni AJ, Eccles JS (1993). Perceived parent-child relationships and early adolescents’ orientation toward peers. Developmental Psychology.

[CR8] Huhman ME, Potter LD, Nolin MJ, Piesse A, Judkins DR, Banspach SW, Wong FL (2010). The influence of the VERB campaign on children’s physical activity in 2002 to 2006. American Journal of Public Health.

[CR9] Khurana A, Bleakley A, Jordan AB, Romer D (2014). The protective effects of parental monitoring and internet restriction on adolescents’ risk of online harassment. Journal of Youth and Adolescence.

[CR10] Livingstone S (2002). Young people and new media: Childhood and the changing media environment.

[CR11] Livingstone, S. (2009). Children and the internet. Polity.

[CR12] Livingstone, S., & Bober, M. (2006). Regulating the Internet at home: Contrasting the perspectives of children and parents. *Digital generations: Children, young people, and new media*, pp. 93–113.

[CR13] Livingstone S, Haddon L (2009). EU Kids Online. Zeitschrift Für Psychologie/Journal of Psychology.

[CR14] Lobe, B., Livingstone, S., Ólafsson, K., & Vodeb, H. (2011). *Cross*-*National Comparison of Risks and Safety on the Internet: Initial analysis from the EU Kids Online survey of European children*.

[CR15] Luna, R., & Finkelhor, D. (1998). *School based prevention programs: Lessons for child victimization prevention*. Retrieved October 21, 2014 from. http://www.unh.edu/ccrc/pdf/CV30.pdf.

[CR16] Mitchell KJ, Jones L, Finkelhor D, Wolak J (2014). Trends in unwanted online experiences and sexting final report.

[CR17] Morrongiello BA, Rennie H (1998). Why do boys engage in more risk taking than girls? The role of attributions, beliefs, and risk appraisals. Journal of Pediatric Psychology.

[CR18] Pasquier, D. (2001). Media at home: Domestic interactions and regulation. *Children and their changing media environment: A European comparative study*, pp. 61–177.

[CR19] Prochaska JO, Johnson SS, Lee P, Schron E, Ockene J, Schumaker S, Exum WM (1998). The transtheoretical model of behavior change. The handbook of behavioral change.

[CR20] Segers E, Verhoeven L (2009). Learning in a sheltered Internet environment: The use of WebQuests. Learning and Instruction.

[CR21] Shapka JD, Law DM (2013). Does one size fit all? Ethnic differences in parenting behaviors and motivations for adolescent engagement in cyberbullying. Journal of Youth and Adolescence.

[CR22] Steinberg L (2004). Risk taking in adolescence: What changes, and why?. Annals of the New York Academy of Sciences.

[CR23] Steinberg L, Cauffman E (1996). Maturity of judgment in adolescence: Psychosocial factors in adolescent decision making. Law and Human Behavior.

[CR25] Swaim RC, Kelly K (2008). Efficacy of a randomized trial of a community and school-based anti-violence media intervention among small-town middle school youth. Prevention Science.

[CR26] Valcke M, De Wever B, Van Keer H, Schellens T (2011). Long-term study of safe internet use of young children. Computers & Education.

[CR27] Valcke M, Schellens T, Van Keer H, Gerarts M (2007). Primary school children’s safe and unsafe use of the internet at home and at school: An exploratory study. Computers in Human Behavior.

[CR28] Van Grinsven, V., Van Der Woud, L., & Elphic, E. (2011). Rapportage Onderzoek Mediawijsheid in het basis-en voortgezet onderwijs. Retrieved from http://files.beeldengeluid.nl/pdf/Educatie_Duo_Eindrapportage_Mediawijsheid_augustus_30-1-2012_2011.pdf.

[CR29] Wishart JM, Oades CE, Morris M (2007). Using online role play to teach Internet safety awareness. Computers & Education.

